# Supplementary material for: Post-Vaccination and Post-Infection Immunity to the Hepatitis B Virus and Circulation of Immune-Escape Variants in the Russian Federation 20 Years after the Start of Mass Vaccination
Source: Vaccines (Basel). 2023 Feb 13;11(2):430. doi: 10.3390/vaccines11020430 (PMC9962567; doi:10.3390/vaccines11020430)
Supplement: Supplementary file 1 [file vaccines-11-00430-s001.zip › vaccines-2129536-supplementary.pdf]

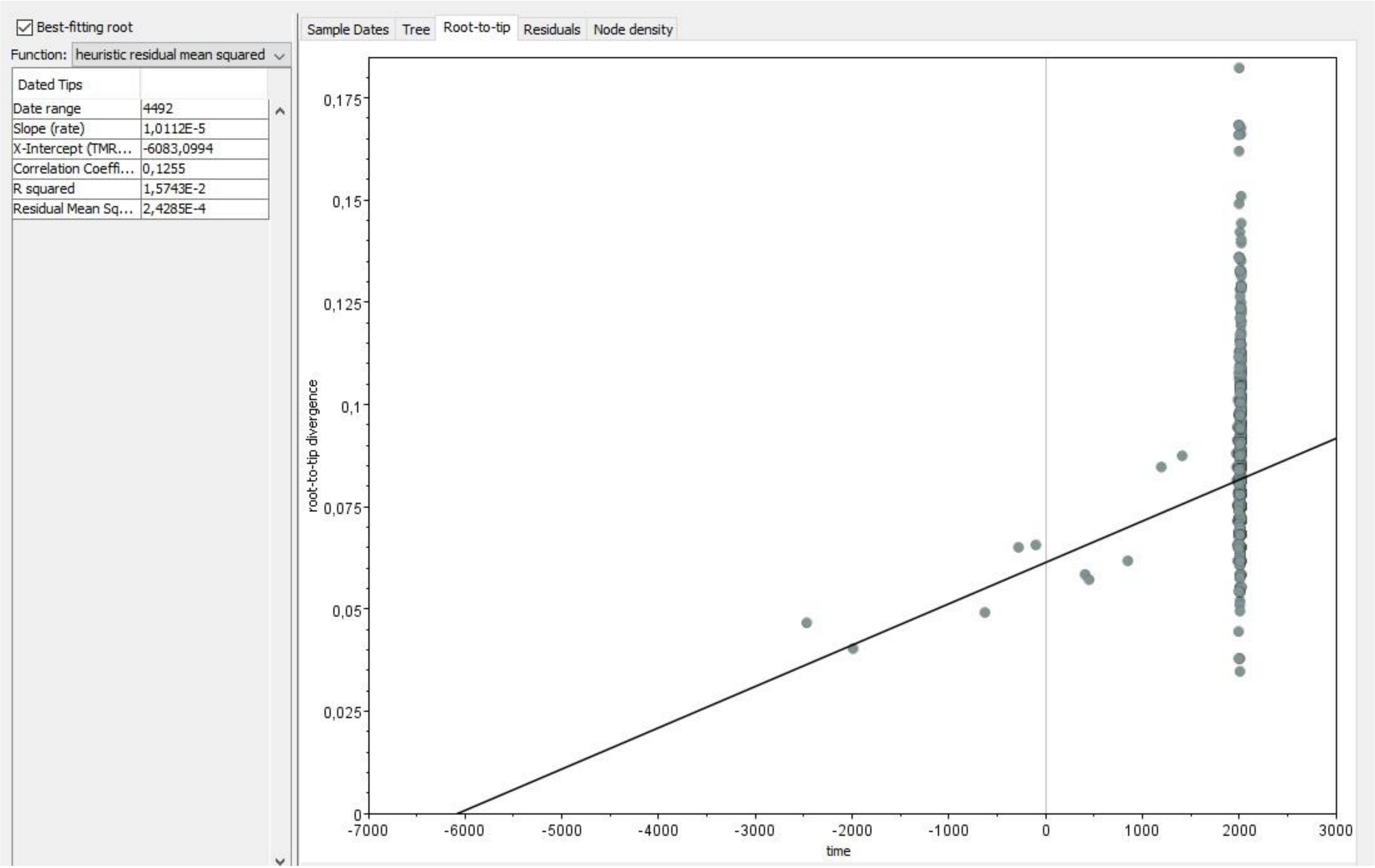

Supplementary figure S1. Temporal signal linear regression graph for HBV 640 nt fragment

Supplementary Table S1. HBsAg detection rates in healthy volunteers

Region	Age groups, years N HBsAg positive/N tested (% [95% CI]) in age cohorts								
	0-9	10-14	15-19	20-29	30-39	40-49	50-59	≥60	All age groups
Kaliningrad Region	0/298 (0%)	0/101 (0%)	0/104 (0%)	1/103 (1,0 [0.0-5.8]%)	2/129 (1.6 [0.1-5.8]%)	0/116 (0%)	1/99 (1.0 [0.0-6.0]%)	0/100 (0%)	4/1050 (0.4 [0.1-1.0]%)
St. Petersburg and Leningrad region	1/353 (0.3 [0.0-1.8]%)	0/113 (0%)	0/129 (0%)	4/1454 (0.3 [0.1-0.7]%)	11/1961 (0.6 [0.3-1.0]%) **	1/668 (0.1 [0.0-0.9]%) **	3/401 (0.7 [0.2-2.3]%)	1/246 (0.4 [0.0-2.5]%)	21/5325 (0.4% [0.2-0.6]%) **
Dagestan Republic	6/1254 (0.5 [0.2-1.1]%) *	4/639 (0.6 [0.2-1.7]%) *	7/545 (1.3 [0.6-2.7]%) **	16/755 (2.1 [1.3-3.4]%) **	38/550 (6.9 [5.1-9.4]%) *;**	11/277 (4 [2.1-7.0]%) **	17/307 (5.5 [3.4-8.7]%) *;**	18/530 (3.4 [2.1-5.3]%) **	117/4857 (2.4 [2.0-2.9]%) **
Moscow and Moscow Region	0/563 (0%)	0/184 (0%)	0/255 (0%)	8/2273 (0.4 [0.2-0.7]%)	12/3106 (0.4 [0.2-0.7]%) **	5/1226 (0.4 [0.1-1.0]%)	4/592 (0.7 [0.2-1.8]%)	2/281 (0.7 [0.0-2.7]%)	31/8480 (0.4 [0.3-0.5]%) **37
Tatarstan Republic	0/133 (0%)	0/58 (0%)	0/86 (0%)	0/103 (0%)	0/110 (0%)	2/107 (1,9 [0.1-7]%)	0/110 (0%)	0/116 (0%)	2/823 (0.24 [0.1-0.94]%)
Sverdlovsk Region	0/172 (0%)	0/61 (0%)	0/204 (0%)	0/51 (0%)	1/147 (0.7 [0.0-4.1]%)	2/102 (2 [0.0-7.3]%)	1/99 (1 [0.0-6.0]%)	2/99 (2 [0.1-7.5]%)	6/935 (0.6 [0.3-1.4]%)
Tuva Republic	1/239 (0.4 [0.0-2.6]%)	0/97 (0%)	0/158 (0%)	0/142 (0%)	2/166 (1.2 [0.0-4.6]%)	1/111 (0.9 [0.0-5.4]%)	8/108 (7.4 [3.6-14.1]%) *;**	2/98 (2 [0.1-7.6]%)	14/1119 (1.2 [0.7-2.1]%)
Novosibirsk Region	1/1395 (0.1 [0.0-0.4]%) *	1/698 (0.1 [0.0-0.9]%)	2/1177 (0.2 [0.0-0.7]%) *	7/1337 (0.5 [0.2-1.1]%)	16/1613 (1.0 [0.6-1.6]%)	12/828 (1.4 [0.8-2.5]%)	9/519 (1.7 [0.9-3.3]%) *	15/756 (2.0 [1.2-3.3]%) *	63/8323 (0.8 [0.6-1.0]%)
Khabarovsk Region	2/1286 (0.2 [0.0-0.6]%) *	1/537 (0.2 [0.0-1.2]%)	0/548 (0%)	1/765 (0.1 [0.0-0.8]%)	14/743 (1.9 [1.1-3.2]%) *	4/391 (1.0 [0.3-2.7]%)	6/394 (1.5 [0.6-3.4]%)	8/573 (1.4 [0.7-2.8]%)	36/5237 (0.7% [0.5-0.9]%)
National average	11/5693 (0.2 [0.1-0.4]%)	6/2488 (0.24 [0.1-0.5]%)	9/3206 (0.28 [0.1-0.5]%)	37/6983 (0.53 [0.4-0.7]%)	96/8525 (1.13 [0.9-1.4]%)	38/3826 (1.0 [0.72-1.4]%)	49/2629 (1.9 [1.4-2.5]%)	48/2799 (1.7 [1.3-2.3]%)	294/36,149 (0.8 [0.7-0.9]%)

* $p<0.05$ (Chi-square with Yates' correction) when compared age-specific values with average value for each region

** $p<0.05$ (Chi-square with Yates' correction) when compared age-specific values with national average age-specific value

Supplementary Table S2. Anti-HBc antibody detection rates in healthy volunteers

Region	Age groups, years<br>N anti-HBc antibody positive/N tested (% [95% CI]) in age cohorts								
	0-9	10-14	15-19	20-29	30-39	40-49	50-59	≥60	All age groups
Kaliningrad Region	37/298<br>(12.4 [9.1-16.7]%)<br>**	19/101<br>(18.8 [12.3-27.6]%)<br>**	13/104<br>(12.5 [7.3-20.3%]%)<br>**	13/103<br>(12.6 [7.4-20.5]%)	20/129<br>(15.5 [10.2-22.8]%)	21/116<br>(18.1 [12.1-26.2]%)	27/99<br>(27.3 [19.4-36.8]%)<br>*	30/100<br>(30.0 [21.9-39.6]%)<br>*	180/1050<br>(17.1 [15.0-19.5]%)**
St. Petersburg and Leningrad region	22/353<br>(6.2 [4.1-9.3]%)<br>*	9/113<br>(8.0 [4.1-14.6]%)	4/129<br>(3.1 [1-8]%)<br>*	90/1454<br>(6.2 [5.1-7.6]%)<br>*;**	163/1961<br>(8.3 [7.2-9.6]%)<br>*;**	109/668<br>(16.3 [13.7-19.3]%)<br>*;**	79/401<br>(19.7 [16.1-23.9]%)<br>*;**	68/246<br>(27.6 [22.4-33.6]%)<br>*	544/5325<br>(10.2 [9.4-11.1]%)<br>**
Dagestan Republic	149/1254<br>(11.9 [10.2-13.8]%)<br>*;**	70/639<br>(11.0 [8.8-13.6]%)<br>*;**	63/545<br>(11.6 [9.1-14.5]%)<br>*;**	127/755<br>(16.8 [14.3-19.7]%)<br>*;**	207/550<br>(37.6 [33.7-41.8]%)<br>*;**	128/277<br>(46.2 [40.4-52.1]%)<br>*;**	139/307<br>(45.3 [39.9-50.9]%)<br>*;**	237/530<br>(44.7 [40.5-49]%)<br>*;**	1120/4857<br>(23.1 [21.9-24.3]%)**
Moscow and Moscow Region	44/563<br>(7.8 [5.9-10.4]%)<br>*	8/184<br>(4.3 [2.1-8.5]%)<br>*	15/255<br>(5.9 [3.5-9.6]%)<br>*	176/2279<br>(7.7 [6.7-8.9]%)<br>*	328/3116<br>(10.5 [9.5-11.7]%)<br>**	197/1239<br>(15.9 [14-18]%)<br>*;**	122/594<br>(20.5 [17.5-24]%)<br>*;**	65/281<br>(23.1 [18.6-28.4]%)<br>*;**	955/8480<br>(11.3 [10.6-12.0]%)<br>**
Tatarstan Republic	12/133<br>(9 [5.1-15.2]%)	3/58<br>(5.2 [1.2-14.7]%)	2/86<br>(2.3 [0.1-8.6]%)<br>*	4/103<br>(3.9 [1.2-10]%)<br>*	20/110<br>(18.2 [12-26.5]%)	18/107<br>(16.8 [10.8-20.1]%)	12/110<br>(10.9 [6.2-18.3]%)<br>**	24/116<br>(20.7 [14.3-29]%)<br>*;**	95/823<br>(11.5 [9.5-13.9]%)<br>**
Sverdlovsk Region	14/172<br>(8.1 [4.8-13.3]%)<br>*	10/61<br>(16.4 [9-27.9]%)<br>**	16/204<br>(7.8 [4.8-12.4]%)<br>*	10/51<br>(19.6 [10.8-32.7]%)<br>**	18/147<br>(12.2 [7.8-18.6]%)	26/102<br>(25.5 [18-34.8]%)<br>*	29/99<br>(29.3 [21.2-38.9]%)<br>*	31/99<br>(31.3 [23-41]%)<br>*	154/935<br>(16.5 [14.2-19.0]%)<br>**
Tuva Republic	43/239<br>(18.0 [13.6-23.4]%)<br>*;**	15/97<br>(15.5 [9.5-24.1]%)<br>*;**	16/158<br>(10.1 [6.2-15.9]%)<br>*	25/142<br>(17.6 [12.2-24.8]%)<br>*;**	67/166<br>(40.4 [33.2-48]%)<br>*;**	65/111<br>(58.6 [49.3-67.3]%)<br>*;**	66/108<br>(61.1 [51.7-69.8]%)<br>*;**	58/98<br>(59.2 [49.3-68.4]%)<br>*;**	355/1119<br>(31.7 [29.1-34.5]%)<br>**
Novosibirsk Region	72/1395<br>(5.2 [4.1-6.5]%)<br>*;**	21/698<br>(3.0 [2-4.6]%)<br>*;**	45/1177<br>(3.8 [2.9-5.1]%)<br>*;**	118/1337<br>(8.8 [7.4-10.5]%)<br>*	193/1613<br>(12.0 [10.5-13.6]%)	184/828<br>(22.2163 [19.5-25.2]%)<br>*	111/519<br>(21.4 [18.1-25.1]%)<br>*;**	234/756<br>(31.0 [27.8-34.3]%)<br>*	978/8323<br>(11.8 [11.1-12.5]%)<br>**117
Khabarovsk Region	67/1286<br>(5.2 [4.1-6.6]%)<br>*;**	18/537<br>(3.4 [2.1-5.3]%)<br>*;**	38/548<br>(6.9 [5.1-9.4]%)<br>*	57/765<br>(7.5 [5.8-9.5]%)<br>*	137/743<br>(18.4 [15.8-21.4]%)<br>*;**	104/391<br>(26.6 [22.5-31.2]%)<br>*	120/394<br>(30.5 [26.1-35.2]%)<br>*	196/573<br>(34.2 [30.4-38.2]%)<br>*	737/5237<br>(14.1 [13.2-15.0]%)
National average	460/5693<br>(8.1 [7.4-8.8]%)	173/2488<br>(7.0 [6.0-8.0]%)	212/3206<br>(6.6 [5.8-7.5]%)	620/6983<br>(8.9 [8.2-9.6]%)	1153/8525<br>(13.5 [12.8-14.3]%)	852/3826<br>(22.3 [21-23.6]%)	705/2629<br>(26.8 [25.2-28.5]%)	943/2799<br>(33.7 [32-35.5]%)	5118/36,149<br>(14.2 [13.8-14.5]%)

* p<0.05 (Chi-square with Yates' correction) when compared age-specific values with average value for each region

** p<0.05 (Chi-square with Yates' correction) when compared age-specific values with national average age-specific value

Supplementary Table S3. Proportion of healthy volunteers reactive for anti-HBs, but non-reactive for anti-HBc

Region	Age groups, years<br>N reactive for anti-HBs, but non-reactive for anti-HBc/N tested (% [95% CI]) in age cohorts								
	0-9	10-14	15-19	20-29	30-39	40-49	50-59	≥60	All age groups
Kaliningrad Region	117/298<br>(39.3 [33.9-44.9]%)<br>*,**	18/101<br>(17.8 [11.5-26.5]%)<br>**	23/104<br>(22.1 [15.2-31.1]%)<br>**	50/103<br>(48.5 [39.1-58.1]%)<br>*,**	38/129<br>(29.5 [22.3-37.9]%)	19/116<br>(16.4 [10.7-24.3]%)<br>*	15/99<br>(15.2 [9.3-23.6]%)<br>*	3/100<br>(3.0 [0.6-8.8]%)<br>*,**	283/1050<br>(27.0 [24.4-29.7]%))<br>**
St. Petersburg and Leningrad region	185/353<br>(52.4 [47.2-57.6]%)<br>*	46/113<br>(40.7 [32.1-49.9]%)	58/129<br>(45.0 [36.7-53.6]%)<br>**	963/1454<br>(66.2 [63.8-68.6]%)<br>*	668/1961<br>(34.1[32-36.2]%)<br>*	136/668<br>(20.4 [17.5-23.6]%)<br>*	66/401<br>(16.5 [13.1-20.4]%)<br>*	27/246<br>(11 [7.6-15.5]%)<br>*	2149/5325<br>(40.4 [39.1-41.7]%)
Dagestan Republic	436/1254<br>(34.8 [32.2-37.5]%)<br>*,**	174/639<br>(27.2 [23.9-30.8]%)<br>**	175/545<br>(32.1 [28.3-36.2]%)	374/755<br>(49.5 [46-53.1]%)<br>*,**	126/550<br>(22.9 [19.6-26.6]%)<br>*,**	42/277<br>(15.2 [11.4-19.9]%)<br>*,**	50/307<br>(16.3 [12.6-20.9]%)<br>*	70/530<br>(13.2 [10.6-16.4]%)<br>*	1447/4857<br>(29,8 [28.5-31.1]%)<br>**
Moscow and Moscow Region	221/563<br>(39.3 [35.3-43.4]%)<br>**	55/184<br>(29.9 [23.7-36.9]%)<br>*	113/255<br>(44.3 [38.4-50.5]%)<br>*,**	1467/2273<br>(64.5 [62.6-66.5]%)<br>*,**	1009/3106<br>(32.5 [30.9-34.2]%)<br>*,**	204/1226<br>(16.6 [14.7-18.8]%)<br>*,**	92/592<br>(15.5 [12.8-18.7]%)<br>*	21/281<br>(7.5 [4.9-11.2]%)<br>*,**	3182/8480<br>(37.5 [36.5-38.5]%)<br>**
Tatarstan Republic	58/133<br>(43.6 [35.5-52.1]%)	24/58<br>(41.4 [29.6-54.2]%)<br>*	22/86<br>(25.6 [17.5-35.8]%)<br>*	56/103<br>(54.4 [44.8-63.7]%)<br>*,**	39/110<br>(35.5 [27.1-44.8]%)	41/107<br>(38.3 [29.7-47.8]%)<br>**	39/110<br>(35.5 [27.1-44.8]%)<br>**	26/116<br>(22.4 [15.7-30.9]%)<br>*,**	305/823<br>(37.1 [33.8-40.4]%)
Sverdlovsk Region	86/172<br>(50.0 [42.6-57.4]%)<br>*	12/61<br>(19.7 [11.5-31.5]%)<br>**	32/204<br>(15.7 [11.3-21.3]%)<br>*,**	26/51<br>(51.0 [37.7-64.1]%)<br>*,**	81/147<br>(55.1 [47-62.9]%)<br>*,**	6/102<br>(5.9 [2.5-12.5]%)<br>*,**	9/99<br>(9.1 [4.7-16.6]%)<br>*,**	7/99<br>(7.1 [3.2-14.1]%)<br>*	259/935<br>(27.7 [24.9-30.7]%)<br>**
Tuva Republic	65/227<br>(28.6 [23.1-34.8]%)<br>*,**	9/96<br>(9.4 [4.8-17.1]%)<br>*,**	12/159<br>(7.5 [4.3-12.8]%)<br>*,**	70/141<br>(49.6 [41.5-57.8]%)<br>*,**	57/166<br>(34.3 [27.5-41.9]%)<br>*	14/112<br>(12.5 [7.5-20]%)<br>*,**	10/108<br>(9.3 [5-16.4]%)<br>*,**	9/98<br>(9.2 [4.7-16.7]%)<br>*	246/1119<br>(22.0 [19.6-24.5]%)<br>**
Novosibirsk Region	772/1395<br>(55.3 [52.7-57.9]%)<br>*,**	349/698<br>(50.0 [46.3-53.7]%)<br>**	436/1177<br>(37.0 [34.3-39.8]%)<br>*	1145/1337<br>(85.6 [83.7-87.4]%)<br>*,**	732/1613<br>(45.4 [43-47.8]%)<br>**	228/828<br>(27.5 [24.6-30.7]%)<br>*,**	111/519<br>(21.4 [18.1-25.1]%)<br>*	117/756<br>(15.5 [13.1-18.2]%)<br>*	3890/8323<br>(46.7 [45.6-47.8]%)<br>**
Khabarovsk Region	763/1286<br>(59.3 [56.6-62]%)<br>*,**	233/537<br>(43.4 [39.3-47.6]%)<br>**	252/548<br>(46.0 [41.9-50.2]%)<br>**	572/765<br>(74.8 [71.6-77.7]%)<br>*,**	320/743<br>(43.1 [39.6-46.7]%)<br>*,**	124/391<br>(31.7 [27.3-36.5]%)<br>*,**	104/394<br>(26.4 [22.3-31]%)<br>*,**	107/573<br>(18.7 [15.7-22.1]%)<br>*,**	2475/5237<br>(47.3 [45.9-48.7]%)<br>**
National average	2703/5693<br>(47.5 [46.2-48.8]%)	920/2488<br>(37.0 [35.1-38.9]%)	1123/3206<br>(35.0 [33.4-36.7]%)	4723/6983<br>(67.6 [66.5-68.7]%)	3070/8525<br>(36.0 [35-37]%)	814/3826<br>(21.3 [20.0-22.6]%)	496/2629<br>(18.9 [17.4-20.4]%)	387/2799<br>(13.8 [12.6-15.2]%)	14,236/36,149<br>(39.4 [38.9-39.9]%)

* $p<0.05$ (Chi-square with Yates' correction) when compared age-specific values with average value for each region

** $p<0.05$ (Chi-square with Yates' correction) when compared age-specific values with national average age-specific value

Supplementary Table S4. Proportion of healthy volunteers non-reactive for either anti-HBs, or anti-HBc

Region	Age groups, years<br>N non-reactive for either anti-HBs, or anti-HBc/N tested (% [95% CI]) in age cohorts								
	0-9	10-14	15-19	20-29	30-39	40-49	50-59	≥60	All age groups
Kaliningrad Region	143/298 (48 [42.4-53.7]%) *	64/101 (63.4 [53.6-72.1]%) *	68/104 (65.4 [55.8-73.9]%)*;**	40/103 (38.8 [30.0-48.5]%)**	71/129 (55.0 [46.4-63.4]%) *	76/116 (65.5 [56.5-73.6]%) *	72/99 (72.7 [63.2-80.6]%)*;**	70/100 (70. [60.4-78.1]%) *;**	604/1050 (57.5 [54.5-60.5]%) **
St. Petersburg and Leningrad region	146/353 (41.4 [36.3-46.6]%) *	58/113 (51.3 [42.2-60.4]%)	67/129 (51.9 [43.4-60.4]%)	401/1454 (27.6 [25.3-29.9]%) *	1130/1961 (57.6 [55.4-59.8]%) *;**	423/668 (63.3 [59.6-66.9]%) *;**	256/401 (63.8 [59.0-68.4]%) *;**	151/246 (61.4 [55.2-67.3]%) *;**	2632/5325 (49.4 [48.1-50.7]%) **
Dagestan Republic	669/1254 (53.3 [50.6-56.1]%) *;**	395/639 (61.8 [58.0-65.5]%) *;**	307/545 (56.3 [52.1-60.4]%) *;**	254/755 (33.6 [30.4-37.1]%) *;**	217/550 (39.5 [35.5-43.6]%) *;**	107/277 (38.6 [33.1-44.5]%) *;**	118/307 (38.4 [33.2-44.0]%) *;**	223/530 (42.1 [37.9-46.3]%) *;**	2290/4857 (47.1 [45.7-48.5]%)
Moscow and Moscow Region	287/563 (51 [46.9-55.1]%) **	121/184 (65.8 [58.6-72.2]%) *;**	127/255 (49.8 [43.7-55.9]%)	632/2279 (27.7 [25.9-29.6]%) *	1775/3116 (57.0 [55.2-58.7]%) *;**	829/1239 (66.9 [64.2-69.5]%) *;**	381/594 (64.1 [60.2-67.9]%) *;**	195/281 (69.4 [63.8-74.5]%) *;**	4347/8480 (51.1 [50.0-52.2]%) **
Tatarstan Republic	62/133 (46.6 [38.4-55.1]%)	31/58 (53.5 [40.8-65.7]%) *	62/86 (72.1 [61.8-80.5]%) *;**	43/103 (41.8 [32.7-51.4]%) **	50/110 (45.5 [36.5-54.8]%)	47/107 (43.9 [34.9-53.4]%) **	59/110 (53.6 [44.4-62.7]%)	66/116 (56.9 [47.8-65.6]%)	420/823 (51.0 [47.6-54.4]%) **
Sverdlovsk Region	72/172 (41.9 [34,7-49,3]%) *	39/61 (63.9 [51,4-74,9]%)	156/204 (76.5 [70,2-81,8]%) *;**	15/51 (29.4 [18,6-43,1]%) *	48/147 (32.7 [25,6-40,6]%) *;**	70/102 (68.6 [59,1-76,8]%) *;**	62/99 (62.6 [52.8-71.5]%)	61/99 (61.6 [51.8-70.6]%)	523/935 (55.9 [52.7-59.1]%) **
Tuva Republic	119/239 (49.8 [3.5-56.1]%)	73/97 (75.3 [65.8-82.8]%) *;**	140/158 (88.6 [82.6-92.8]%) *;**	46/142 (32.4 [25.2-40.5]%) *	43/166 (25.9 [19.8-33.1]%) *;**	32/111 (28.8 [21.2-37.9]%) *;**	32/108 (29.6 [21.8-38.9]%) *;**	30/98 (30.6 [22.3-40.4]%) *;**	515/1119 (46.0 [43.1-49.0]%)
Novosibirsk Region	551/1395 (39.5 [37.0-42.1]%) **	328/698 (47.0 [43.3-50.7]%) *;**	431/1177 (36.6 [33.9-39.4]%) *;**	339/1337 (25.4 [23.1-27.8]%) *	688/1613 (42.7 [40.3-45.1]%) **	416/828 (50.2 [46.8-53.6]%) *;**	297/519 (57.2 [52.9-61.4]%) *	405/756 (53.6 [50.0-57.1]%) *	3455/8323 (41.5 [40.4-42.6]%) **
Khabarovsk Region	457/1286 (35.5 [33.0-38.2]%) *;**	286/537 (53.3 [49.0-57.4]%) *	259/548 (47.3 [43.1-51.5]%) *	136/765 (17.8 [15.2-20.7]%) *;**	286/743 (38.5 [35.1-42.0]%) **	163/391 (41.7 [36.9-46.6]%) **	170/394 (43.1 [38.4-48.1]%) **	270/573 (47.1 [43.1-51.2]%) *;**	2027/5237 (38.7 [37.4-40.0])% **

National average	2506/5693 (44.0 [42.7-45.3]%)	1395/2488 (56.1 [54.1-58.0]%)	1617/3206 (50.4 [48.7-52.2]%)	1906/6983 (27.3 [26.3-28.4]%)	4308/8525 (50.5 [49.5-51.6]%)	2163/3826 (56.5 [55.0-58.1]%)	1447/2629 (55.0 [53.1-56.9]%)	1471/2799 (52.6 [50.7-54.4]%)	16,813/36,149 (46.5 [46.0-47.0]%)

* $p<0.05$ (Chi-square with Yates' correction) when compared age-specific values with average value for each region

** $p<0.05$ (Chi-square with Yates' correction) when compared age-specific values with national average age-specific value
